# Homocysteine Triggers Inflammatory Responses in Macrophages through Inhibiting CSE-H_2_S Signaling via DNA Hypermethylation of CSE Promoter

**DOI:** 10.3390/ijms160612560

**Published:** 2015-06-03

**Authors:** Jiao-Jiao Li, Qian Li, Hua-Ping Du, Ya-Li Wang, Shou-Jiang You, Fen Wang, Xing-Shun Xu, Jian Cheng, Yong-Jun Cao, Chun-Feng Liu, Li-Fang Hu

**Affiliations:** 1 Department of Neurology, Jiangsu Key Laboratory of Translational Research and Therapy for Neuro-Psycho-Diseases, the Second Affiliated Hospital of Soochow University, Soochow University, Suzhou 215004, China; E-Mails: dragonrabbit@163.com (J.-J.L.); duhuaping226@126.com (H.-P.D.); yjyali@sina.com (Y.-L.W.); 0319503013@163.com (S.-J.Y.); wangfen_1982@126.com (F.W.); xingshunxu@hotmail.com (X.-S.X.); yongjuncao@126.com (Y.-J.C.); 2 Institute of Neuroscience, Soochow University, Suzhou 215123, China; E-Mails: liqian90626@163.com (Q.L.); jiancheng8@hotmail.com (J.C.); 3 Department of Pharmacology, School of Pharmaceutical Science, Soochow University, Suzhou 215123, China

**Keywords:** homocysteine, cystathionine γ-lyase, hydrogen sulfide, macrophage, DNA methylation

## Abstract

Hyperhomocysteinemia (HHcy) is an independent risk factor of atherosclerosis and other cardiovascular diseases. Unfortunately, Hcy-lowering strategies were found to have limited effects in reducing cardiovascular events. The underlying mechanisms remain unclear. Increasing evidence reveals a role of inflammation in the pathogenesis of HHcy. Homocysteine (Hcy) is a precursor of hydrogen sulfide (H_2_S), which is formed via the transsulfuration pathway catalyzed by cystathionine β-synthase and cystathionine γ-lyase (CSE) and serves as a novel modulator of inflammation. In the present study, we showed that methionine supplementation induced mild HHcy in mice, associated with the elevations of TNF-α and IL-1β in the plasma and reductions of plasma H_2_S level and CSE expression in the peritoneal macrophages. H_2_S-releasing compound GYY4137 attenuated the increases of TNF-α and IL-1β in the plasma of HHcy mice and Hcy-treated raw264.7 cells while CSE inhibitor PAG exacerbated it. Moreover, the *in vitro* study showed that Hcy inhibited CSE expression and H_2_S production in macrophages, accompanied by the increases of DNA methyltransferase (DNMT) expression and DNA hypermethylation in *cse* promoter region. DNMT inhibition or knockdown reversed the decrease of CSE transcription induced by Hcy in macrophages. In sum, our findings demonstrate that Hcy may trigger inflammation through inhibiting CSE-H_2_S signaling, associated with increased promoter DNA methylation and transcriptional repression of *cse* in macrophages.

## 1. Introduction

Homocysteine (Hcy) is a non-essential sulfhydryl-containing amino acid derived from methionine metabolism, and is dynamically regulated via two metabolic pathways: remethylation and transsulfuration pathway, respectively. Elevation in plasma Hcy, also called hyperhomocysteinemia (HHcy), commonly occurs due to high methionine diet, dietary deficiencies of folic acid/vitamin B, mutations in genes encoding Hcy-metabolizing enzymes, and impaired renal clearance as well [1]. Although severe HHcy (>100 µM) is rare, mild (15–30 µM) to moderate (31–100 µM) HHcy occurs in 5%–10% of the general population. HHcy is an independent risk factor of cardiovascular disease, and also associated with the onset of several neurologic disorders such as stroke, mild cognitive impairment and Alzheimer’s disease [2]. However, the mechanisms by which HHcy exert toxic effects remain elusive. Multiple pathogenesis, chronic inflammation in vascular bed in particular, is implicated in the progression of HHcy-associated diseases.

Hydrogen sulfide (H_2_S) has long been stigmatized as a toxic gas for centuries. In 1996 it was recognized as a neuromodulator and gasotransmitter, exhibiting great relevance in regulating multiple pathophysiological processes in mammals [3,4]. Indeed, H_2_S is one of the end products of the transsulfuration pathway. It can be produced from homocysteine and cysteine by synthases including cystathionine β-synthase (CBS), cystathionine γ-lyase (CSE), and 3-mercaptopyruvate sulfurtransferase in combination with cysteine aminotransferase [5]. CBS controls the transsulfuration of Hcy to cystathionine, which is subsequently converted into α-ketobutyrate and l-cysteine by CSE. l-cysteine can be further metabolized to H_2_S. Recently, H_2_S was found to exert cytoprotective effects in HHcy-induced cell damage in heart, brain and kidney [6,7,8]. Hcy-lowering strategies such as supplementation with folate and B vitamins had disappointingly limited effects in reducing cardiovascular and cerebrovascular events [9,10]. This led us to hypothesize that long-term exposure to HHcy may suppress H_2_S generation via the transsulfuration pathway and that HHcy-associated pathogenesis may be linked to the impairment in H_2_S production.

Given that monocyte/macrophage-mediated chronic inflammation is greatly implicated in HHcy-associated pathogenesis and that H_2_S serves as a modulator of inflammation [11,12], in this study we sought to examine the effect and underlying mechanisms of Hcy on H_2_S generation in raw264.7 cells and the peritoneal macrophages isolated from the mice with mild HHcy. Our findings revealed that Hcy repressed CSE transcription in macrophages by inducing DNA hypermethylation in *cse* promoter, decreased H_2_S production and thus caused the elevations in pro-inflammatory cytokines generation in macrophages and in the plasma of mice with HHcy.

## 2. Results and Discussion

### 2.1. Effects of Methionine Administration on Plasma Hcy, H_2_S and Pro-Inflammatory Cytokine Levels in C57BL/6 Mice and CSE Expression in Peritoneal Macrophages

**Figure 1:**
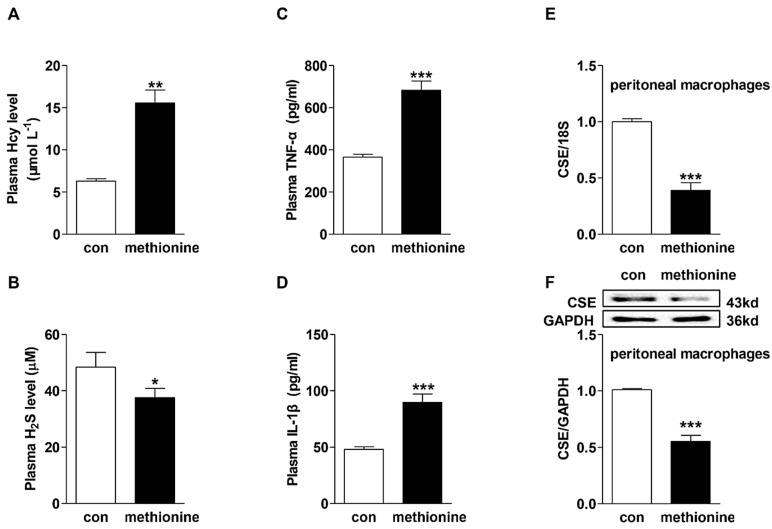
Plasma homocysteine (Hcy) (**A**), hydrogen sulfide (H_2_S) (**B**) and cytokines levels (**C**,**D**), as well as cystathionine γ-lyase (CSE) mRNA (**E**) and protein expressions (**F**) in the peritoneal macrophages of C57BL/6 mice with or without methionine-supplementation. The measurements of Hcy, H_2_S and cytokines level were performed as described in Materials and Methods. CSE mRNA level was examined by quantitative PCR while its protein expression determined by immunoblotting analysis. 18S mRNA and glyceraldehyde-3-phosphate dehydrogenase (GAPDH) protein served as loading controls. Results are presented as mean ± SEM, *n* = 4–7 in each group. * *p <* 0.05, ** *p <* 0.01, *** *p <* 0.001 compared to control groups.

Dietary supplementation with 2% methionine in drinking water resulted in significant elevations in plasma Hcy (15.56 ± 1.553 µM in methionine-treated group *versus* 6.3 ± 0.298 µM in control group) and pro-inflammatory cytokine (TNF-α, IL-1β) levels, accompanied by the reduction in plasma H_2_S level in C57BL/6 mice (Figure 1A–D). Moreover, the CSE mRNA and protein expressions decreased approximately by 60% and 45%, respectively, in the peritoneal macrophages isolated from the methionine-treated mice compared to control dieted mice.

### 2.2. Effects of H_2_S on Cytokines Level in the Plasma of Methionine Diet-Treated Mice and Hcy-Treated Raw264.7 Macrophages

**Figure 2:**
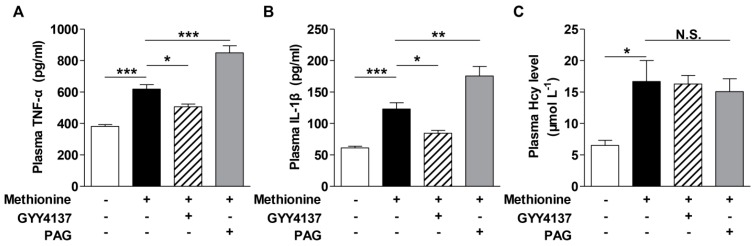

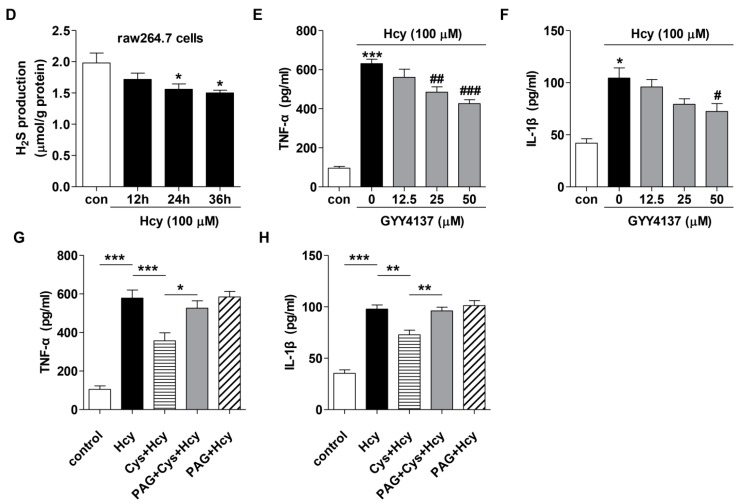
Effect of H_2_S modulation on the pro-inflammatory cytokines in the plasma of methionine-treated mice and Hcy-treated raw264.7 cells. (**A**–**C**) H_2_S-releasing agent GYY4137 ameliorated while CSE inhibitor PAG aggravated the increases of plasma TNF-α (**A**) and IL-1β (**B**) levels in methionine diet-treated mice. Neither GYY4137 nor PAG altered the plasma Hcy level (**C**); For methionine diet, C57BL/6 mice were administered with 2% methionine in drinking water for 14 consecutive days. For GYY4137 and PAG treatment, mice were pre-treated with GYY4137 (50 mg/kg/day, i.p.) or PAG (37.5 mg/kg/day, i.p.) for three days before subjected to 2% methionine supplement. *n* = 4–7 animals in each group; (**D**) Hcy treatment reduced H_2_S production in raw264.7 macrophages; (**E**,**F**) GYY4137 (12.5, 25, 50 µM) pre-treatment for 1 h attenuated the increases of TNF-α and IL-1β in Hcy (100 µM, 24 h)-treated macrophages; (**G**,**H**) Raw264.7 cells were pre-treated with Cys (1 mM), in the presence or absence of PAG (1 mM) co-treatment for 30 min, before being subjected to Hcy (100 µM, 24 h) treatment. *n* = 4–9. * *p <* 0.05, ** *p <* 0.01, *** *p <* 0.001 compared to its corresponding control group. ^#^ *p <* 0.05, ^##^ *p <* 0.01, ^###^ *p <* 0.001 compared to Hcy group. N.S., not significant.

To determine whether H_2_S down-regulation was related to the elevations of pro-inflammatory cytokines in the mice with mild HHcy, methionine-fed mice were injected with H_2_S-releasing agent GYY4137 and CSE inhibitor DL-propargylglycine (PAG). As can be seen from Figure 2A,B, GYY4137 (50 mg/kg/day, i.p.) co-treatment led to a marked decrease of TNF-α and IL-1β levels in the plasma of methionine-treated mice, while PAG (37.5 mg/kg/day, i.p.) aggravated the increases of these cytokines level. Neither GYY4137 nor PAG had any influence on the plasma Hcy level (Figure 2C) induced by methionine diet.

The observations were confirmed in raw264.7 cells. As shown in Figure 2D–H, 100 µM Hcy reduced the H_2_S production but enhanced TNF-α and IL-1β generation. H_2_S-releasing agent GYY4137 co-treatment attenuated the Hcy-induced elevations of TNF-α and IL-1β production in a dose-dependent manner. Pre-treatment with H_2_S precursor cysteine (Cys, 1 mM) in raw264.7 cells also attenuated the increases of TNF-α and IL-1β generation, both of which could be reversed by the addition of PAG (1 mM). Co-treatment with PAG, in the absence of Cys, did not obviously affect the cytokines level in Hcy-treated cells. These results indicate the down-regulation of CSE-H_2_S generation may be involved in Hcy-induced elevations of pro-inflammatory cytokines in both the plasma and macrophage.

### 2.3. Hcy Suppressed CSE Expression in Hcy-Treated Raw264.7 Cells

**Figure 3:**
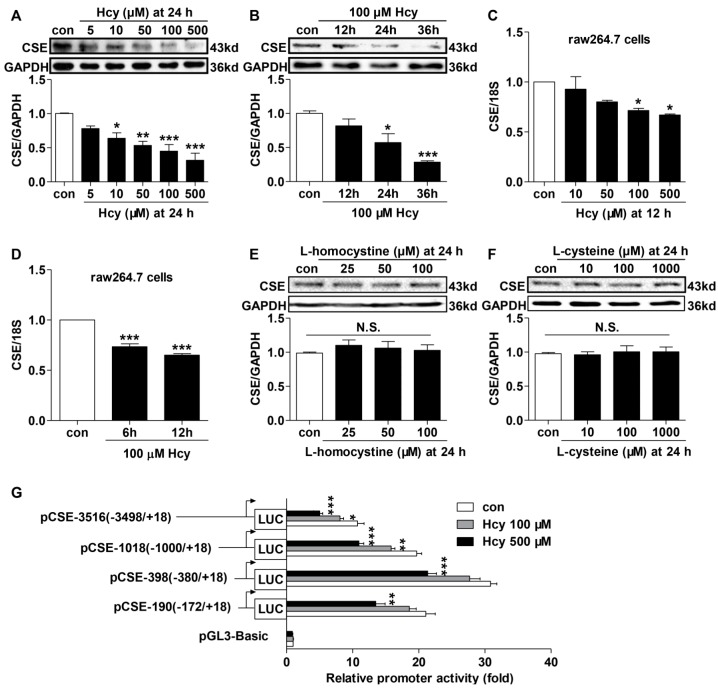
Effects of Hcy on CSE expression and promoter activity in raw264.7 macrophages. (**A**–**D**) Hcy down-regulated CSE mRNA and protein expressions in raw264.7 macrophages in dose- and time-dependent manners (**E**,**F**). Effect of l-homocystine and l-cysteine on CSE protein expression. CSE protein expression was evaluated by immunoblotting analysis while CSE mRNA level determined by quantitative PCR. GAPDH protein and 18S mRNA served as loading controls. Mean ± SEM, *n* = 3–8. (**G**) Reporter assay of a series of different fragments of *cse* promoter activity in raw264.7 cells that were treated with 100 or 500 µM Hcy for 12 h. Luciferase activities were expressed as fold increases over pGL3-basic vector. *n* = 7. * *p <* 0.05, ** *p <* 0.01, *** *p <* 0.001 compared to its corresponding control group. N.S., not significant.

We then examined the effect of Hcy on CSE expression in macrophages. It was observed that treatment with increasing concentrations of Hcy (5, 10, 50, 100 and 500 µM) resulted in marked decreases of CSE protein and mRNA expression in a dose-dependent manner (Figure 3A,C). Moreover, Hcy time-dependently reduced CSE protein and mRNA levels in raw264.7 cells (Figure 3B,D). In contrast, neither l-homocystine (the oxidized form of l-homocysteine) nor l-cysteine (a reducing thiol) had any significant effect on CSE protein expression (Figure 3E,F). The promoter activities of different fragments of mouse *cse* promoter were further evaluated with luciferase assay (Figure 3G). Compared with pGL3-basic vector, the pGL3-CSE construct (−3498/+18) transfection enhanced the luciferase activity by about 10-fold. Deletion from −3498 to −381 resulted in an additional 20-fold increase, indicating a possibility that negative regulatory elements for *cse* transcription may exist in this region. However, a deletion from −380 to −173 markedly reduced the promoter activity, indicating a basal and essential element for *cse* transcription may be located between −380 and +18. Compared with controls, treatment with 100 µM Hcy reduced the promoter activities to varying degrees, and 500 µM Hcy resulted in a more pronounced repression. This implies that Hcy treatment may repress CSE transcription in macrophages.

### 2.4. Hcy up-Regulated DNMT Expression and Enhanced DNA Hypermethylation in cse Promoter

**Figure 4:**
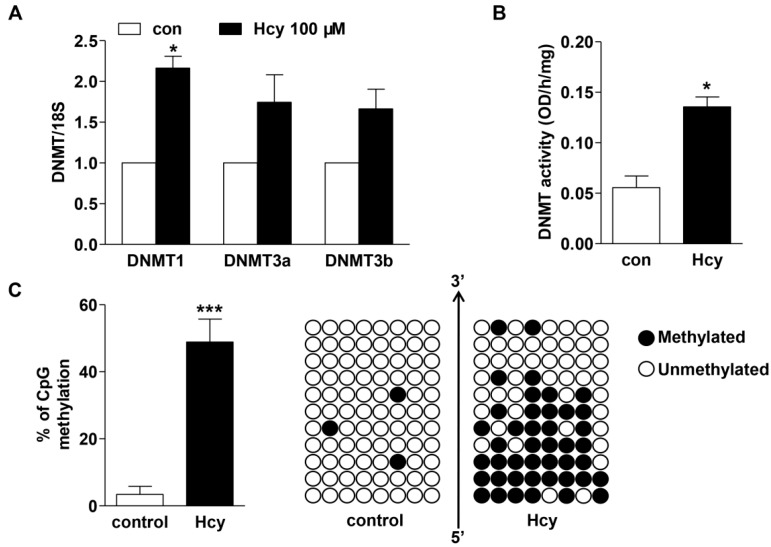
Hcy enhanced DNMT expression and activity in macrophages, accompanied with DNA hypermethylation in *cse* gene promoter. (**A**,**B**) One hundred micromoles Hcy treatment for 6 h enhanced DNMTs mRNA expression (**A**) and activity (**B**) in raw264.7 cells. *n* = 3; (**C**) BSP sequencing revealed DNA hypermethylation of mouse *cse* promoter that contains 11 CpG sites in Hcy-treated macrophages. Each column indicated the clones from Control (**left**) and Hcy (100 µM, **right**)-treated cells. The methylated CpG sites were labeled in black circles and unmethylated CpG sites were labeled in white circles. * *p <* 0.05, *** *p <* 0.001 compared to its corresponding control.

Next, we focused on the mechanisms that may underlie the repression by Hcy on *cse* transcription. Interestingly, it was observed that accompanying with the decrease in CSE expression, 100 µM Hcy treatment markedly increased the DNMT1 mRNA level (Figure 4A) and DNMT activity (Figure 4B) in raw264.7 cells, although DNMT3a and DNMT3b mRNA expression were merely marginally elevated. This prompted us to examine whether DNMT upregulation may drive DNA methylation in *cse* gene promoter and thus repress its transcription. To achieve this, raw264.7 cells were treated with 100 µM Hcy or vehicle. Genomic DNA samples of vehicle- and Hcy-treated cells were then treated with sodium bisulfite and the 215 bp (−417/−203) fragment containing 11 CpG sites were amplified by PCR, followed by DNA sequencing (Figure 4C). The percentage of DNA methylation in the 11 CpG sites was found to be significantly enhanced in Hcy-treated cells compared to control group.

### 2.5. DNMT1 Inhibition Reversed the Repression of Hcy on CSE Transcription

**Figure 5:**
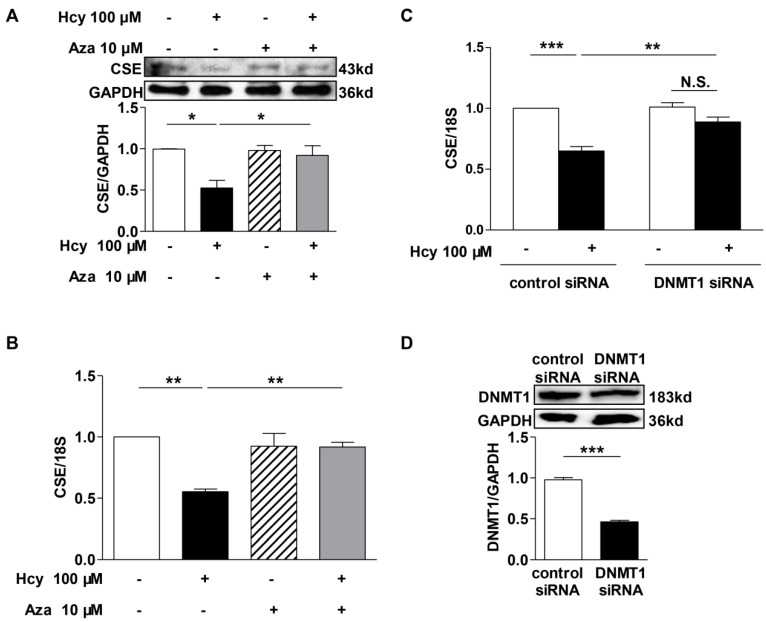
DNMTs inhibition reversed the repression of Hcy on CSE expression. DNMTs inhibition with its inhibitor Aza (**A**,**B**) or DNMT1 siRNA (**C**) almost reversed the decreases of CSE expression induced by Hcy treatment. The knockdown efficiency of DNMT1 siRNA was determined by immunoblotting analysis (**D**). *n* = 3–4. * *p <* 0.05, ** *p <* 0.01, *** *p <* 0.001. N.S., not significant.

To confirm whether DNMT1 was responsible for DNA hypermethylation of *cse* gene in Hcy-treated macrophages, we studied the expression of CSE protein and mRNA in DNMT1-suppressed cells. As shown in Figure 5, Hcy caused about 50% reduction of CSE protein and mRNA levels, which were reversed by DNMT inhibitor 5-Aza-2′-deoxycytidine (Aza) co-treatment (Figure 5A,B). Moreover, DNMT1 knockdown with siRNAs also alleviated the decrease of *cse* transcription caused by Hcy treatment, compared with scrambled siRNA-transfected cells. One hundred micromoles Hcy failed to significantly reduce CSE mRNA expression in DNMT1-deficient cells (Figure 5C). The knockdown efficiency of DNMT1 siRNA reached about 50% as determined by its protein expression (Figure 5D).

### 2.6. Discussion

In the present study, mild HHcy associated with elevations of pro-inflammatory cytokines TNF-α and IL-1β in the plasma, was induced in mice by 2% methionine supplement in drinking water for two weeks. We demonstrated that accompanying with the increase in plasma Hcy level, the plasma H_2_S level and CSE expression in the peritoneal macrophages isolated from HHcy mice were markedly decreased. H_2_S-releasing compound GYY4137 attenuated the increase of TNF-α and IL-1β in both the plasma and Hcy-treated macrophages while CSE inhibitor PAG exacerbated it, although neither GYY4137 nor PAG altered the Hcy level in plasma. Moreover, we showed that Hcy inhibited the transcription and protein expression of CSE and H_2_S production in time- and dose-dependent manners in raw264.7 macrophage cells. In addition, Hcy up-regulated the expression and activity of DNMT, associated with the increase of DNA methylation in mouse *cse* promoter. Suppression of DNMT with its inhibitor or siRNA reversed the decrease of CSE mRNA induced by Hcy in macrophages. Taken together, our findings suggest that the impairment in CSE-H_2_S production in macrophages may be responsible for HHcy-induced vascular inflammation in mice, and that DNA hypermethylation of CpG rich region in *cse* promoter may contribute to the down-regulation of CSE transcription and thus the decrease in H_2_S production.

So far, the exact cellular and molecular mechanisms that underlie HHcy-associated exacerbation of cardiovascular and other diseases remain elusive. Emerging evidence indicates that monocyte/macrophage-derived inflammatory responses may be involved [13]. Our findings also demonstrated the elevations of pro-inflammatory cytokines in the plasma of 2% methionine-treated mice with mild HHcy. Although it may be difficult to determine whether inflammation is an upstream or downstream event in HHcy-associated pathogenesis, the impairment in H_2_S production may underlie the elevations of plasma cytokines level in HHcy mice based on our *in vitro* and *in vivo* findings. This is consistent with the recent notion that H_2_S is a novel and endogenous modulator of inflammation [14].

**Figure 6:**
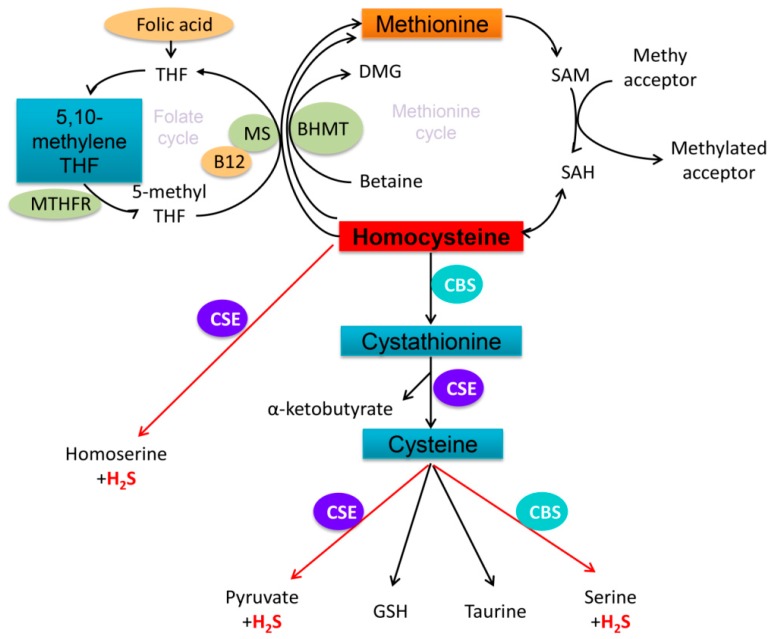
Schematic representations of mammalian homocysteine metabolism. Homocysteine metabolism is at the intersection of two main pathways: remethylation and transsulfuration pathway. Intracellular folate cycling is tightly connected to the remethylation pathway, in which methionine is activated by condensation with adenosine triphosphate (ATP) to yield the ubiquitous methyl donor, SAM, which is transformed into SAH by donating its methyl group to the substrates of methylation reactions. Subsequently, SAH gives rise to homocysteine in a reversible reaction that favors SAH over homocysteine production. The transsulfuration process is catalyzed by two vitamin B_6_-dependent enzymes: CBS and CSE. Hcy can be sequentially converted into cystathionine and then cysteine by CBS and CSE and subsequently results in the generation of H_2_S and other by-products including homoserine, pyruvate, GSH, taurine, and serine as well. MS, methionine synthase; THF, tetrahydrofolate; MTHFR, methylene tetrahydrofolatereductase; BHMT, betaine-homocysteine *S*-methyltransferase; DMG, dimethylglycine; SAM, *S*-adenosyl methionine; SAH, *S*-adenosylhomocysteine; CBS, cystathionine β-synthase; CSE, cystathionine γ-lyase; GSH, glutathione.

CBS and CSE play important roles in Hcy metabolism and H_2_S synthesis, as summarized in Figure 6. On the one hand, Hcy can be sequentially converted into cystathionine and then cysteine by CBS and CSE, respectively. Both CBS and CSE can use cysteine as substrates for H_2_S synthesis. On the other hand, Hcy can be directly converted by CSE into homoserine and H_2_S. Therefore, it is conceivable that deficiency in either *cbs* or *cse*, or vitamin B_6_ may result in HHcy and decrease H_2_S production in the blood. CBS deficiency may lead to HHcy [15]. However, the role of CSE in HHcy and its associated pathogenesis is poorly understood. In this study, we showed that the plasma Hcy level was inversely related to H_2_S level in methionine-treated mice, which is absent from any genetic or Vitamin B_6_ deficiency. The observations were confirmed in our *in vitro* study, and consistent with a recent report that Hcy reduced H_2_S production in mouse glomerular mesangial cells [16]. Furthermore, we provided the evidence that the down-regulation of CSE may be responsible for the decrease of H_2_S production in Hcy-treated cells and mice with HHcy. In addition, our study demonstrated that Hcy enhanced DNA methylation in *cse* promoter and repressed its transcription in macrophages. In fact, CSE is predominantly expressed in the heart, vascular tissues and peripheral monocyte/macrophages, and serves as a major source of H_2_S production in the cardiovascular and immune systems. The relevance of CSE in HHcy-associated cardiovascular diseases should not be underestimated. Our study not only reveals a novel epigenetic regulation of CSE transcription, but also implies that disruption of H_2_S biosynthesis and homeostasis may be an effector of HHcy-associated pathogenesis. Thus, modulation of H_2_S may become a potential therapeutic strategy for HHcy-related disorders. Actually, H_2_S was recently reported to inhibit myocardial injury [6], decrease colitis severity [17] and attenuate neurodegeneration and neurovascular dysfunction [8] in rodent models associated with HHcy. Moreover, a most recent study by Christopher Hine *et al.* [18] revealed that endogenous H_2_S production is essential for dietary restriction benefits. Hence, it would be of great relevance to monitor the plasma H_2_S level in patients with HHcy.

Surely, plasma H_2_S may be contributed by various tissues and cells, apart from monocytes/macrophages. For example, liver, heart and kidney, as well as endothelial cells were reported to express H_2_S synthases and produce H_2_S *in vivo* [19,20]. In the current study, we focused on the contribution of macrophages merely, because inflammation is mainly involved in HHcy-associated pathogenesis and that monocytes/macrophages serve as the most important type of cells that mediates inflammation. More importantly, it was reported that alterations in DNA methylation profiles were already present in the peripheral blood monocytes of four-week-old ApoE^−/−^ mice with no detectable atherosclerotic lesion [21]. The relationship between HHcy and H_2_S in other tissues/cells warrants further study in the future.

The transcriptional regulation of CSE is poorly understood. DNA methylation, driven by DNMTs, is important epigenetic machinery that affects gene expression. DNA demethylation facilitates gene transcription, whereas hypermethylation represses. There are three subtypes DNMT1, DNMT2 and DNMT3. They all use *S*-adenosyl methionine as a source of methyl groups and transfer the methyl to the 5′-position of cytosine residues of CpG dinucleotides [22]. DNMT1, the most abundant DNMT, has high affinity for hemimethylated DNA and maintain the constitutive methylation status of DNA. DNMT2 is expressed at substantially lower levels in adult tissues and its role in methylation remains unclear. DNMT3a and DNMT3b are mainly responsible for *de novo* methylation, and show no preference for hemimethylated over unmethylated DNA substrates [23]. Our study showed that the CpG rich region in *cse* promoter was hypermethylated after Hcy treatment, associated with significant increases in DNMT expression and activity. Moreover, the prediction results from CpG Island Searcher (Available online: http://www.urogene.org) showed there exists a 116 bp (−121/−6) of CpG island in the murine CSE promoter region. Due to the high ratio of CpG dinucleotides, the 190 bp (−172/+18) fragment of mouse *cse* gene promoter may also be modified by methylation although the bisulfite sequencing assay in our study did not include the DNA sequence in this fragment. More importantly, DNMT1 inhibition or knockdown alleviated the repression by Hcy on CSE transcription, indicating that DNA hypermethylation may contribute to the CSE down-regulation in Hcy-treated macrophages and in mice with mild HHcy.

Both global and gene-specific alterations in DNA methylation are associated with abnormal phenotypes in diseases [24]. Elevated Hcy levels were reported to alter global DNA methylation and also induce promoter specific methylation in many genes implicated in atherosclerosis and other disorders [9,25,26]. In the present study, we demonstrated for the first time that Hcy induced DNA hypermethylation and repressed *cse* transcription. Further, we showed that the reduction in CSE expression and H_2_S production in macrophages may be involved in HHcy-induced vascular inflammation, which is implicated in both the initiation and progression of atherosclerosis and other vascular diseases. This is consistent with a recent study that *cse* deficiency accelerated the progression of atherogenesis in ApoE^−/−^ mice. In addition, a recent study by Chang *et al.* [27] also reported that Hcy disrupted the growth and survival of endothelial cells through a G protein-mediated pathway associated with altered promoter DNA methylation and the transcriptional repression of fibroblast growth factor-2 [28]. Thus, our findings supported the recent notion that long-term exposure to HHcy may have “memory effect” on specific genes via epigenetic machinery that eventually affects the expression profiles of these disease-related genes. The reason for HHcy-induced gene-specific effects remains unclear. In fact, Hcy mainly exists in the oxidized form as l-homocystine in the blood owing to the high reactivity of its thiol group. However, l-homocystine did not have similar effects on CSE expression as Hcy did. Moreover, another reducing thiol l-cysteine had no significant effects on CSE expression either. These results indicate that the inhibition on CSE expression and H_2_S generation might be Hcy specific. Moreover, numerous *in vitro* studies have shown that Hcy could induce biological changes such as mitochondrial toxicity, oxidative stress and inflammation in different types of cells or tissues [29,30,31].Therefore, it is Hcy rather than homocystine or other thiols that may be responsible for HHcy-associated pathogenesis.

## 3. Experimental Section

### 3.1. Reagents and Antibodies

GYY4137 was obtained from Cayman Chemical (Ann Arbor, MI, USA). Methionine, PAG, l-Homocysteine, l-homocystine, l-cysteine, Aza, and other chemicals unless specified were purchased from Sigma (St. Louis, MO, USA). Mouse anti-CSE (Abnova H00001491-M01, Taipei, Taiwan), anti-Dnmt1 (Abnova MAB0079, Taipei, Taiwan) and anti-GAPDH (CMCTAG, AT0002, San Diego, CA, USA) were used for immunoblotting analysis. Reagents for cell culture were obtained from Life Technologies (Carlsbad, CA, USA).

### 3.2. Cell Culture

Murine macrophage raw264.7 cells were cultured in Dulbecco’s modified Eagle medium (DMEM) supplemented with 10% fetal bovine serum (FBS), 3.7 g/L NaHCO_3_ and 1% penicillin/streptomycin in a 37 °C incubator with 5% CO_2_. Primary peritoneal macrophage was isolated as we previously reported [14]. In brief, peritoneal cavity of euthanized C57BL/6 mouse was washed with ice-cold DMEM containing 10% FBS to harvest peritoneal cells. After centrifugation, cells were re-suspended in DMEM and seeded in culture dishes. Non-adherent cells were rinsed off at 2 h later, and the adherent cells were used for experimentation after at least 24 h culture.

### 3.3. Animals

Eight-week-old male C57BL/6 mice, obtained from HFK Bio-Technology Co., Ltd. (Beijing, China), were randomly divided into four groups: (1) Control group, fed with normal chow diet; (2) Methionine-treated group, fed with normal chow diet plus 2% (*w*/*v*) methionine in drinking water for 14 days; (3) Methionine plus GYY4137 group, pre-treated with GYY4137 (50 mg/kg/day, i.p.) for 3 days, followed by co-treatment with 2% methionine for 14 days; and (4) Methionine plus PAG group, pre-treated with PAG (37.5 mg/kg/day, i.p.) for 3 days, followed by 2% methionine supplement. Mice were housed under standard conditions (SPF-grade animal room, 12 h light/dark cycle, 24 ± 1 °C, and 70% ± 4% relative humidity). Procedures were in accordance with the guidelines established by the Institutional Animal Care and Use Committee of Soochow University. Plasma Hcy level was determined by Hcy assay kits (Fosun Diagnostics, Shanghai, China).

### 3.4. H_2_S Measurement

To detect the plasma H_2_S level, 75 μL plasma were added sequentially with 475 μL distilled water, 250 μL 1% *w*/*v* zinc acetate, 133 μL *N*,*N*-dimethyl-*p*-phenylenediamine sulfate (20 mM in 7.2 M HCl) and 133 μL FeCl_3_ (30 mM in 1.2 M HCl). The mixture was placed at room temperature for 10 min for color development, followed by adding with 250 μL 10% *w*/*v* trichloroacetic acid to stop reaction. After a brief centrifugation at 4000 rpm for 3 min, 200 μL mixtures were aliquoted and then detected at 668 nm using a microplate reader (Infinite M200 PRO, Grodig, Austria). For blood collection, mice were subjected to fasting for at least 8 h after the last injection with the tested compounds. Blood was collected from the heart and stored in the EDTA-K_2_ anti-coagulant tubes. After centrifugation at 3000 rpm for 5 min, plasma was collected and placed on ice before H_2_S measurement, as described above.

The H_2_S level in the culture supernatants were measured as we previously reported [32]. In brief, cells were cultured in phenol red free DMEM and stimulated with Hcy for 12, 24 and 36 h before culture supernatants were collected. Then, 400 μL supernatants were added sequentially with 4 μL NaOH (1 M), 40 μL *N*,*N*-dimethyl-*p*-phenylenediamine sulfate (20 mM in 7.2 M HCl) and 40 μL FeCl_3_ (30 mM in 1.2 M HCl). After that, the mixtures were incubated at room temperature for 20 min. The formed methylene blue was detected at 668 nm. The H_2_S concentration was assessed using a standard curve of NaHS and normalized by the protein concentration of the cell lysates.

### 3.5. Immunoblotting Analysis

Cells were harvested and lysed in ice-cold lysis buffer (150 mM NaCl, 25 mM Tris, 5 mM EDTA, 1% Nonidet P-40, pH 7.5) with protease inhibitor cocktail tablets (Roche Diagnostics, Penzberg, Germany). Equal amounts of protein lysates were boiled and separated by 10% sodium dodecyl sulfate-polyacrylamide gels and transferred onto nitrocellulose membranes. Membranes were blocked with Tris buffered saline/Tween 20 (TBST) containing 5% (*w*/*v*) non-fat dry milk at room temperature for 1 h, and then incubated overnight at 4 °C with optimized diluted primary antibodies. After that, membranes were washed three times with TBST and incubated with horseradish peroxidase-conjugated secondary antibodies for another 1 h on the shaker. Finally, membranes were washed with TBST and visualized with ECL chemiluminescence (Thermo Company, West Chester, PA, USA). Densitometric analysis was performed with Image J software (National Institute of Health, Bethesda, MD, USA).

### 3.6. Quantitative Polymerase Chain Reaction (PCR)

Total RNA of raw264.7 cells was extracted with Trizol reagent (Invitrogen, Carlsbad, CA, USA) while that of peritoneal macrophages was extracted using an E.Z.N.A.^®^ HP Total RNA Kit (Omega Bio-Tek, Norcross, GA, USA) according to the manufacturer’s instructions. Equal amounts (1 μg) of RNA were reverse-transcribed into cDNA using Revert Aid First Strand cDNA synthesis kit (Fermentas, Beijing China). Quantitative PCR was performed using SYBR Green PCR Master Mix (Invitrogen) on the ABI 7500 system (Applied Biosystems, Foster City, CA, USA). The cycle time values were normalized to 18S of the same sample. The mRNA levels were presented as the relative values to controls. The primers are listed as follows: mouse CSE (forward: 5′-CTTGCTGCCACCATTACG-3′; reverse: 5′-TTCAGATGCCACCCTCCT-3′), Dnmt1 (forward: 5′-GGAGCCCAGCAAAGAGTA-3′; reverse: 5′-GGGAGACACCAGCCAAAT-3′), Dnmt3a (forward: 5′-CACCTATGGGCTGCTGCGAAGAC-3′; reverse: 5′-GGCGGCCAGTACCCTCATAAAGT-3′), Dnmt3b (forward: 5′-CCCACCCAAGCGCCTCAAGACAAA-3′; reverse: 5′-TGGCAGCGCTGAGGGAGGCACATA-3′) and 18S (forward: 5′-TCAACACGGGAAACCTCAC-3′; reverse: 5′-CGCTCCACCAACTAAGAAC-3′).

### 3.7. Cse Promoter Activity Analyses

A *cse*-luciferase reporter containing 3.5 kb genomic DNA fragment of the mouse *cse* promoter (spanning from −3498 to +18 relative to the transcription start site) [20] was a kind gift from Isao Ishii (Keio University, Tsuruoka, Japan). pGL3-CSE-190 (−172 to +18), pGL3-CSE-398 (−380 to +18) and pGL3-CSE-1018 (−1000 to +18) luciferase reporter plasmids were sub-cloned from the 3.5 kb *cse* promoter (−3498 to +18). For promoter activity assay, cells that reached 80% confluence in 24-well plates were transfected with 800 ng pGL3-basic (Promega, Beijing China) or equimolar pGL3-CSE constructs using lipofectamine 2000 (Invitrogen, Carlsbad, CA, USA). One hundred nanograms pSV-β-Galactosidase Vector (Promega), whose activity is unaffected by Hcy, was co-transfected as an internal control. At 24 h post-transfection, cells were treated with 100 or 500 μM Hcy for 12 h before harvest. Luciferase activity was then measured using Steady-Glo^®^ Luciferase Assay System (Promega, E2510). Transfection efficiency was normalized by β-galactosidase activity (Promega, E2000). The promoter activity was expressed as fold increases over pGL3-basic vector transfected group.

### 3.8. Bisulfite Sequencing (BSP) Assay

The methylation status was determined by bisulfite sequencing as previously reported [23]. Genomic DNA extracted by QIAamp DNA mini kit was modified with bisulfite reagents according to the manufacturer’s instructions (QIAGEN, Shanghai, China). This modification resulted in a conversion of unmethylated cytosine to thymine, whereas the methylated cytosine remained unaltered. A total of 20 ng bisulfite-modified DNA was subjected to PCR amplification, and PCR products were cloned into the pMD™18-T vector (TAKARA, Dalian China). At least 8 individual clones were harvested, followed by DNA sequencing by Sangon Biotech (Shanghai, China). The following primers were designed to amplify CpG-rich regions within *cse*: forward, 5′-GATAAGGTGTAAAATTAGGTTAAAGTTA-3′; reverse, 5′- CCCCTTTATCTACAAAATAAAAAC-3′.

### 3.9. Dnmt Activity

Dnmt activity was determined using EpiQuik DNMT Activity/Inhibition Assay Ultra Kit (Epigentek, NY, USA). In brief, the nuclear extracts were prepared using the EpiQuik Nuclear Extraction Kit, and the protein concentrations were determined by BCA protein assay. The amount of methylated DNA was proportional to the DNMT enzymatic activity, which was detected with anti-methyl CpG binding antibody and quantified by a microplate reader at 450 nm. DNMT activity was calculated according to the following formula: DNMT activity (OD/h/mg) = 1000 × (sample OD − blank OD)/(μg protein × initial incubation time in hours).

### 3.10. siRNA Transfection

The small-interfering RNAs (siRNA) targeting mouse Dnmt1 (5′-GGGAGAAAUUAAACUUACUTT-3′ and 5′-AGUAAGUUUAAUUUCUCCCTT-3′) and scrambled siRNAs were synthesized by GenePharma (Shanghai, China). The siRNAs were transfected using lipofectamine 2000 (Invitrogen, Carlsbad, CA, USA). The knockdown efficiency was determined at 12 h post-transfection with immunoblotting analysis.

### 3.11. Cytokine Production Assay

The cytokines levels in plasma and cell culture supernatant were determined by enzyme linked immunoabsorbent assay (ELISA). The contents of TNF-α were determined with ELISA kits from R&D systems (Minneapolis, MN, USA) while that of IL-1β was determined with ELISA kits from BOSTER (Wuhan, China). The absorbance was determined at 450 nm using the microplate reader as described above.

### 3.12. Statistical Analysis

All data are presented as mean ± SEM. Statistical analysis was performed by SPSS Version 19.0 software (SPSS, Chicago, IL, USA) using Student’s *t* test or one-way analysis of variance followed by Tukey *post hoc* analysis where applicable. Differences with *p* < 0.05 were considered statistically signiﬁcant.

## 4. Conclusions

Our findings demonstrate that down-regulation of CSE expression and H_2_S production may contribute to HHcy-triggered inflammation both *in vitro* and *in vivo*, and reveal that DNA hypermethylation may be responsible for Hcy-induced inhibition on CSE expression in macrophages. Our work may be helpful for the understanding of the pathogenesis of HHcy-associated diseases, and lead to new therapeutic strategies based on modulation of H_2_S production.
